# Nadir Creatinine in Congenital Anomalies of the Kidney and Urinary Tract (CAKUT): A Single-Center Experience

**DOI:** 10.3390/children11080928

**Published:** 2024-07-31

**Authors:** Marius-Cosmin Colceriu, Paul Luchian Aldea, Bogdan Bulată, Dan Delean, Alexandra Sevastre-Berghian, Simona Clichici, Andreea-Liana Boț (Răchişan), Teodora Mocan

**Affiliations:** 1 Discipline of Physiology, Department of Functional Biosciences, “Iuliu Hațieganu” University of Medicine and Pharmacy, 400006 Cluj-Napoca, Romania; berghian.alexandra@umfcluj.ro (A.S.-B.); sclichici@umfcluj.ro (S.C.); teodora.mocan@umfcluj.ro (T.M.); 2 Second Pediatric Discipline, Department of Mother and Child, “Iuliu Haţieganu” University of Medicine and Pharmacy, 400177 Cluj-Napoca, Romania; andreea_rachisan@elearn.umfcluj.ro; 3 Discipline of Public Health and Management, Department of Community Medicine, “Iuliu Haţieganu” University of Medicine and Pharmacy, 400006 Cluj-Napoca, Romania; paul.luch.aldea@elearn.umfcluj.ro; 4 Pediatric Nephrology, Dialysis and Toxicology Clinic, Emergency Clinical Hospital for Children, 400177 Cluj-Napoca, Romania; bogdan.bulata@yahoo.com (B.B.); ddelean2003@yahoo.com (D.D.); 5 Nanomedicine Department, Regional Institute of Gastroenterology and Hepatology, 400158 Cluj-Napoca, Romania

**Keywords:** CAKUT, nadir creatinine, children, ESRD

## Abstract

Background/Objectives: Congenital anomalies of the kidney and urinary tract (CAKUT) are the main cause of chronic kidney disease (CKD) requiring renal replacement therapy (RRT) in children, being the leading cause (50–70%) of end-stage renal disease (ESRD) in children and young adults. Our study aimed to assess the natural evolution of various antenatally diagnosed renal malformations and to identify potential prognostic factors to guide the therapeutic management of patients with CAKUT. Methods: We conducted a retrospective study on 205 children with CAKUT. For each patient, analyzing their medical records, we established the nadir value of serum creatinine, defined as the lowest creatinine level during the first year of life. We assessed the value of nadir creatinine as a prognostic marker in patients with CAKUT, and using an ROC curve, we also determined a threshold value of nadir creatinine that predicted progression to ESRD. Results: The male-to-female ratio was 2.8 to 1. The mean gestational age at detection was 29.85 weeks (±6.71). A total of 36 patients (17.6%) had impaired renal function, of which 8 (3.9% of the total) progressed to ESRD. The mean nadir creatinine in patients with ESRD was 1.39 mg/dL. A nadir creatinine cut-off of 0.98 mg/dL had high sensitivity and specificity in identifying patients with progression to ESRD, with an AUC of 0.95 and a 95% confidence interval between 0.86 and 1.05 mg/dL. Conclusions: Our results support the value of nadir creatinine in predicting progression to ESRD, consistent with previously published data.

## 1. Introduction

The group of congenital anomalies of the kidney and urinary tract (CAKUT) comprises a broad spectrum of structural disorders that appear during the embryological development of the reno-urinary tract [1]. The incidence of CAKUT in live and stillborn neonates ranges from 0.3 to 1.6 per 1000, with higher incidence observed in individuals with a family history of renal disease, cancer, or diabetes. About 10% of patients with CAKUT have a positive family history of renal abnormalities. Of all prenatally detected anomalies, CAKUT represents about 20 to 30 percent [2,3,4]. CAKUT is the main cause of chronic kidney disease (CKD) requiring renal replacement therapy (RRT) in children, being the main cause (50–70%) of end-stage renal disease (ESRD) in children and young adults [5,6]. Ureteropelvic junction obstruction (UPJ) is the most common malformation, accounting for 50% of total CAKUT. Other malformations are represented by renal agenesis, kidney dysplasia and hypoplasia, a horseshoe kidney, a multicystic dysplastic kidney (MCDK), a duplex collecting system, an ectopic ureter, a megaureter, vesicoureteral reflux (VUR), and posterior urethral valves (PUV). Some of these anomalies may appear in association with other renal or extrarenal malformations. There are more than 200 syndromes that are associated with CAKUT [1,7,8].

The clinical manifestations of CAKUT vary widely, from asymptomatic cases discovered incidentally to severe forms presenting with renal failure, hypertension, or recurrent urinary tract infections. Early detection and accurate diagnosis are crucial for managing these conditions and preventing long-term renal impairment. Diagnostic approaches typically include prenatal ultrasound, which can identify many anomalies before birth, and postnatal imaging studies, such as renal ultrasound, a voiding cystourethrogram (VCUG), magnetic resonance imaging, and nuclear medicine scans [1,8]. Most patients are diagnosed early in the prenatal period or within the first few months of life. Fetal ultrasound can identify CAKUT in 60% to 85% of individuals, with the highest detection rates occurring in the third trimester. However, some cases are only discovered after birth, often when a child presents with a urinary tract infection (UTI) that leads to further imaging of the kidneys and urinary tract. Individuals born with a reduced number of nephrons, either in one kidney or both, may not experience any symptoms until their teenage years or later, when they might develop hypertension or CKD [2,3].

The natural history of CAKUT is currently incompletely known, which makes early management of the condition challenging. Furthermore, there is a physiological increase in the glomerular filtration rate (GFR) from birth to two years of age, due to the developmental and physiologic renal adjustments to the extra-uterine environment. Thus, managing severe CAKUT in infants should consider and optimize renal development to preserve nephron reserve and extend individual renal function [9,10,11]. Inadequate management at the time of diagnosis of CAKUT can allow progressive degradation of renal function, which can lead to significant morbidity and mortality. The evolution towards CKD stages 2–5 can cause growth retardation, multisystemic complications, and sexual function impairment, with severe physical and psycho-social complications. Therefore, studying the natural evolution of CAKUT and identifying prognostic factors are crucial for proper patient management [2,10,12].

The prognosis of CAKUT varies widely depending on the specific anomaly and its severity. Factors influencing prognosis include the degree of renal function impairment at diagnosis, associated anomalies, and the effectiveness of early intervention. Some conditions, like mild VUR, may resolve spontaneously, while others, like bilateral MCKD, may lead to CKD with impaired renal function or ESRD if not managed appropriately. Management strategies are tailored to the individual patient, based on the specific anomaly, and may include pharmacological treatment, surgical correction, and regular monitoring of renal function. For instance, cases of PUV may require surgical correction to prevent kidney damage, while VUR may be managed with antibiotics and regular surveillance. In recent years, there has been growing interest in identifying predictive biomarkers, such as nadir creatinine levels, that can guide treatment decisions and predict long-term outcomes [1,2,12].

Our study aimed to assess the natural evolution of various renal malformations and to identify potential prognostic factors to guide the therapeutic management of patients with CAKUT. We performed a retrospective study on a group of patients diagnosed antenatally with CAKUT, for which we evaluated the predictive value of nadir creatinine regarding the evolution toward ESRD. In addition, we assessed the influence of gestational age at diagnosis and type of malformation on the prognosis of these patients.

## 2. Materials and Methods

We conducted a retrospective study on a group of 205 children with CAKUT, who were followed up in the Department of Pediatric Nephrology of the Emergency Clinical Hospital for Children, Cluj-Napoca, Romania, over four years (August 2019–July 2023). All the patients included in the study were antenatally detected as having CAKUT, confirmed by postnatal imaging. The fetal ultrasound aspects that were considered suggestive of CAKUT were unilateral or bilateral hydronephrosis, renal cystic dysplasia, and oligohydramnios associated with renal structural changes. An anteroposterior diameter of the renal pelvis greater than 4 mm before the 28th week of gestation and greater than 7 mm after that was considered abnormal [13]. After birth, the patients entered the records of our department, the diagnosis of CAKUT being established by imaging methods such as ultrasonography (US), a voiding cystourethrogram (VCUG), or magnetic resonance urography (UroMRI). The renal malformations evaluated in our study were classified into six categories, represented by unilateral and bilateral VUR, PUV, MCDK, UPJ, and other obstructive uropathies (OU). In the last three categories, we included both patients with unilateral and bilateral involvement. We excluded patients with coexisting malformations, except for patients with PUV and secondary VUR. The latter were included in the category of patients with PUV, this being the primary condition. We included in the study all the patients with CAKUT admitted to our hospital who had at least one positive fetal ultrasound.

We used medical records to obtain data regarding the dynamic values of serum creatinine, surgical interventions, and the evolution of the patients. For each patient, we established the nadir value of serum creatinine, which was defined as the lowest creatinine level during the first year of life. Serum creatine concentration was measured by spectrophotometry, using a Mindray SAL 6000 analyzer (KONELAB; Mindray Bio-Chemical Electronics Co., Ltd., Shenzhen, China). We calculated the estimated glomerular filtration rate (eGFR) using the Schwartz pediatric bedside formula: k × length in cm/serum creatinine in mg/dL (k = 0.413 for children > 1 year, k = 0.45 for full-term infants ≤ 1 year, and k = 0.33 for premature infants ≤ 1 year) [14]. Based on the eGFR, we established the corresponding stage of CKD (1–5) for patients over 2 years of age. Following the 2012KDIGO clinical guidelines for approximating the eGFR reduction in patients under the age of 2, we used age-related normal values provided by Schwartz and Furth as references. These patients were classified as having normal, moderately reduced, or severely reduced age-adjusted eGFR [15]. We considered patients over 2 years of age with chronic renal function impairment as CKD stages 2–5 and those under the age of 2 as having moderately or severely reduced eGFR. Children with CKD stage 5 and in need of RRT were included as having ESRD.

All obtained data were compiled into a database created using Microsoft Office Excel Version 16.78.3 and subsequently analyzed with IBM SPSS Statistics Version 20. For the continuously distributed variables, we applied descriptive statistics to calculate the averages and standard deviations. To assess the statistical relevance of these variables, we utilized Student’s *t*-test. To assess the statistical significance of the correlations between the qualitative variables, we utilized the Pearson Chi-square test. Additionally, we calculated the Pearson correlation coefficient (Pearson r) and created graphical representations of the regression line to illustrate these correlations. For analyzing the relationships between the qualitative variables and continuous numeric variables, we applied the analysis of variance (ANOVA) method. This helped in understanding the associations and variations among these types of variables. The predictive accuracy of nadir creatinine as a prognostic indicator was evaluated using receiver operating characteristic (ROC) curves. We assessed the area under the curve (AUC) values and corresponding confidence intervals to determine the effectiveness of nadir creatinine in classifying disease status. The cut-off value for this assessment was chosen based on the Youden Index, which is recognized as the index for maximum potential effectiveness of a biomarker. We interpreted AUC values in the range of 0.7 to 0.8 as indicative of satisfactory accuracy, whereas AUC values greater than 0.8 were considered to reflect good accuracy of the proposed test [16]. Across all analyses, a *p*-value of less than 0.05 was deemed statistically significant. For *p*-values less than 0.01, we considered the statistical significance to be good, and for *p*-values less than 0.001, the statistical significance was regarded as extremely important, indicating a very high level of confidence in the results with an error margin of just 0.1%.

## 3. Results

A total of 205 children with antenatally suspected CAKUT were enrolled in the study. Out of the total, 152 (74.1%) were boys and 53 (25.9%) were girls, resulting in a male-to-female ratio of 2.8 to 1. The most common type of CAKUT in our study was UPJ, representing almost half of the cases (46%), followed by OU with 16%. PUV and bilateral VUR each represented 13% of the total number of cases. The fewest cases were unilateral VUR (8%) and MCDK (4%).

The mean gestational age at CAKUT feature detection was 29.85 weeks (±6.71). The mean gestational ages at which each malformation was detected are shown in Table 1.

**Figure 1:**
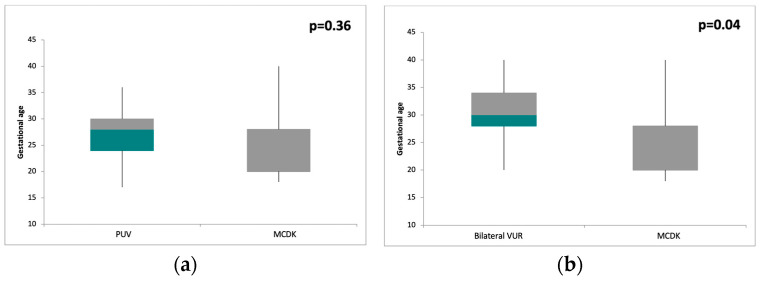
Comparison between gestational ages at detection for different types of CAKUT: (**a**) no significant difference between PUV and MCDK; (**b**) statistically significant difference between bilateral VUR and MCDK.

The values ranged from 24.55 to 30.68 weeks, with MCDK having the lowest value and UPJ having the highest. The ANOVA test indicated that there were statistically significant differences in the gestational ages at detection among the CAKUT types (*p* < 0.05). Similar values were obtained for PUV and MCDK, without significant differences between the gestational ages at detection (Figure 1a). The post hoc analysis revealed the following significant pairwise differences, all with *p*-values less than 0.05: PUV vs. UPJ, PUV vs. unilateral VUR, MCDK vs. bilateral VUR (Figure 1b), MCDK vs. unilateral VUR, and MCDK vs. UPJ. No statistically significant differences were found in the mean gestational ages among bilateral VUR, unilateral VUR, UPJ, and OU.

In our study group, 169 (82.4%) patients had no impaired renal function, based on eGFR being staged as CKD 1. The other 36 (17.6%) patients had impaired renal function, of which 8 (3.9% of the total) were classified as ESRD. Out of the eight patients, four (50%) had PUV, two (25%) had bilateral VUR, and two (25%) had OU as the etiology of ESRD. The mean nadir creatinine in the patients with ESRD was 1.39 mg/dL for those with PUV, 0.76 mg/dL for those with VUR, and 1.18 mg/dL for those with OU. The etiology of CKD with impaired renal function was as follows: PUV 36%, VUR 27.8%, OU 22.2%, MCDK 8.4%, and UPJ 5.6%.

**Figure 2:**
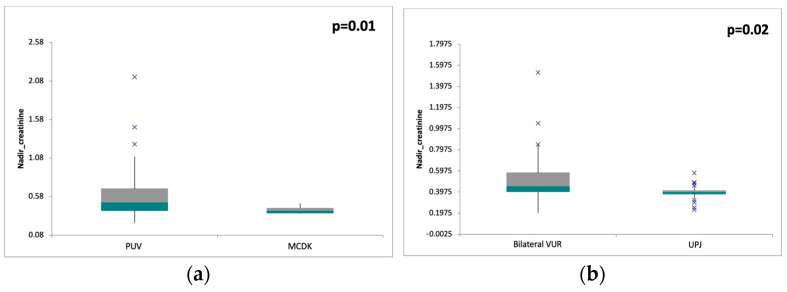
Comparison between nadir creatinine in different types of CAKUT: (**a**) statistically significant difference between PUV and MCDK; (**b**) statistically significant difference between bilateral VUR and UPJ.

For each malformation, we determined the mean value of nadir creatinine, the results being presented in Table 1. The lowest values were recorded for UPJ and MCDK, with 0.39 mg/dL and 0.4 mg/dL, respectively. At the opposite pole were the values obtained in the patients with PUV and bilateral VUR (0.62 mg/dL and 0.52 mg/dL respectively). The ANOVA test revealed statistically significant differences in nadir creatinine levels between different types of CAKUT (*p* < 0.05). The comparative analysis indicated significant differences in nadir creatinine values between MCDK and PUV (Figure 2a) and between UPJ and bilateral VUR (Figure 2b).

**Figure 3:**
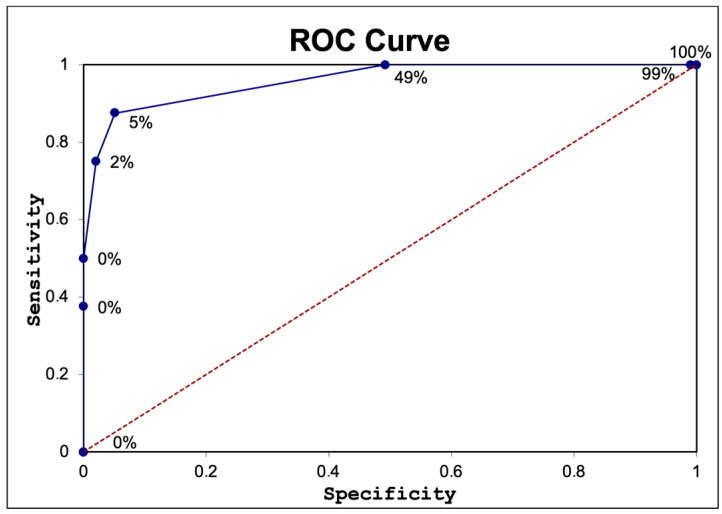
ROC curve for assessment of the accuracy of nadir creatinine in predicting ESRD.

To assess the accuracy of nadir creatinine in predicting ESRD, we utilized a ROC curve. Figure 3 shows that a nadir creatinine cut-off of 0.98 mg/dL had high sensitivity and specificity in identifying patients with progression to ESRD. Nadir creatinine was an outstanding predictive test with an AUC of 0.95 and a 95% confidence interval between 0.86 and 1.05 mg/dL. The statistical significance was extremely important (*p* < 0.001).

## 4. Discussion

Through the present study, we aimed to analyze the pediatric population with CAKUT in our hospital and assess the utility of nadir creatinine as a prognostic marker.

The epidemiological assessment of the 205 patients in our study revealed a male predominance of CAKUT, with a male-to-female ratio of 2.8 to 1. Previously published studies also supported male predominance in CAKUT patients. Thus, in a study conducted on 524 newborns with CAKUT, Melo et al. reported a male-to-female ratio of 3.3 to 1 [17]. Scott et al. published the results of a study on 725 patients, in which the gender distribution was 66.6% males and 33.4% females, with a male-to-female ratio of 2 to 1 [18]. Bondagji et al. reported a male-to-female ratio of 2.1 to 1 in a study on 128 children with CAKUT [11]. Another study with similar results was conducted by Richter-Rodie et al. They examined 309 patients with CAKUT, 63.6% of whom were boys [13]. An important note regarding sex distribution is that certain pathologies, such as PUV, occur only in boys. This is one of the key factors that explains the predominance of the male sex among patients with CAKUT.

The most common type of CAKUT diagnosed in our patients was UPJ, constituting nearly half of the total cases (46.3%). UPJ was followed by VUR, found in 20.4% of patients, with 12.6% having bilateral VUR. In descending order of frequency followed OU, PUV, and MCDK, constituting 15.6%, 13.2%, and 4.4% respectively. This frequency distribution is in agreement with the data reported in previous studies [19]. The most frequently observed renal anomaly found on fetal ultrasound is hydronephrosis. It can indicate conditions such as UPJ, OU, PUV, or VUR. Richter-Rodier et al. found that in a study of 309 patients with CAKUT, hydronephrosis was present in 74.8% of the cases, 5.5% of the cases had a urethral malformation, such as PUV, and only 8% of the patients had VUR [13]. In a similar study, Choi et al. found that 61.7% of antenatally diagnosed CAKUT cases had hydronephrosis [20]. Isac et al. surveyed 252 patients with CAKUT. They found that 52% had UPJ, 16.26% had VUR, 8.7% had MCDK, and 7.93% had lower urinary tract malformations, including PUV [21]. The differences between the reported results were due to the inclusion of conditions such as a solitary kidney, a horseshoe kidney, or a duplex kidney in their studies.

The patients enrolled in our study had urinary tract anomalies detected prenatally via ultrasonography and confirmed by postnatal imaging. The mean gestational age at detection was 29.85 weeks, with a standard deviation of 6.71 weeks. The earliest detection was at 16 weeks, while the latest detection was at 40 weeks. In their study, Bondagji et al. recorded a mean gestational age of 26 weeks at the time of diagnosis, ranging from 18 to 36 weeks [11]. A study conducted by Danziger et al. on 71 patients diagnosed with severe CAKUT revealed that the median age of detection was 21.3 weeks of gestation [3].

Our study demonstrated that the timing of prenatal detection for CAKUT varied depending on the nature and severity of the condition. The earliest detection was recorded for MCDK, with a mean gestational age of 24.55 weeks. This early detection is attributed to the distinct structural alterations in the renal parenchyma observable on fetal ultrasound. MCDK is characterized by multiple non-functional cysts within the kidney, which replace normal renal tissue [11,13]. A similar gestational age at detection, with no statistically significant difference, was obtained in patients with PUV (27.03 weeks). PUV is a condition involving obstruction of the urethra, leading to bilateral dilation of the renal collecting system and possible damage to the renal parenchyma. The bilateral nature and potential impact on the entire reno-urinary system facilitate early detection through fetal ultrasound [13,20].

Statistically significant higher gestational ages at detection were recorded in the patients with VUR, OU, and UPJ, with mean values of 30.53, 30.62, and 30.68 weeks, respectively. During the antenatal period, the primary ultrasound alteration observed in these patients was the occurrence of hydronephrosis. Pathologies that lead to structural changes in the renal parenchyma, such as MCDK, and those with bilateral involvement that may decrease the renal parenchymal index, like PUV, can be detected much earlier by fetal ultrasound. This is in contrast to conditions that primarily cause dilation of the urinary tract, such as VUR, OU, or UPJ, which are detected later [13,20].

Thus, the gestational age of detection of ultrasound abnormalities could be a tool for diagnostic guidance on the type of urinary malformation. If certain types of CAKUT are consistently associated with earlier or later gestational ages, it could alert clinicians to apply the proper prenatal care and clinical management. The information might adjust clinical strategies, like planning intrauterine or postpartum surgical interventions, or initiating antibiotic prophylaxis immediately after birth, and also allow immediate postnatal interventions to prevent further complications. Understanding GA variations might help stratify risk levels, guide the frequency and nature of follow-up appointments, and help in parental counseling on potential postnatal interventions or monitoring needs. Early detection allows for a more coordinated approach involving pediatric nephrologists, urologists, and obstetricians to ensure comprehensive care planning adapted to each type of CAKUT.

Renal function impairment, classified as CKD stages 2–5 in patients over 2 years and as moderately or severely reduced eGFR in those under 2 years, was recorded in 36 patients (17.5%). PUV emerged as the leading cause of CKD in our study, accounting for 36% of cases. The highest mean nadir creatinine level recorded in our study was in the PUV patients (0.62 mg/dL), highlighting the severe impact of PUV on renal function. These findings are consistent with previous research indicating that PUV is a major contributor to renal dysfunction in pediatric populations [6,12]. VUR and OU together determined the alteration of renal function in 18 children, representing half of the patients with moderately or severely reduced eGFR. This aligns with studies demonstrating the high prevalence of renal scarring and CKD progression in children with VUR [22,23] and the detrimental effects of obstructive uropathy on renal health [12,21]. Our study found that bilateral VUR patients had a mean nadir creatinine level of 0.52 mg/dL, while those with OU had a level of 0.48 mg/dL. Other CAKUT conditions, such as unilateral VUR and MCDK, showed lower mean nadir creatinine levels, with UPJ presenting the lowest values. These findings suggest a relatively milder impact on renal function compared to PUV and bilateral VUR, corroborating existing literature that reported similar trends [10,11]. Notably, eight patients in our study progressed to ESRD, accounting for 3.9% of the total cohort. This progression underscores the potential severity of CAKUT and its impact on long-term renal outcomes [15]. It highlights the necessity for proactive monitoring and individualized management strategies to prevent or delay the onset of ESRD in affected patients. Half of the ESRD patients had PUV and the other half had bilateral VUR and OU in equal percentages. The highest mean nadir creatinine value was recorded in the patients with PUV, followed by OU and bilateral VUR. We found that over 85% of the CKD with impaired renal function cases and the highest nadir creatinine values were associated with bilateral involvement, where the sterile pressure effect of the urine on the kidney tissue was present. High-pressure sterile urine can adversely affect renal parenchyma by inducing local ischemia, which triggers the release of toxic metabolites, proinflammatory cytokines, and reactive oxygen species. This initiates a cascade of local inflammation that leads to architectural remodeling and fibrosis. Metalloproteinases play a significant role in this process, contributing to diffuse parenchymal lesions and abnormal renal development. Additionally, the elevated pressure of sterile urine causes glomerular injury, resulting in secondary proteinuria that further exacerbates the local inflammatory response. Moreover, there is evidence of altered prostaglandin synthesis in this context. Thus, abnormal development of the renal structures will determine the impairment of renal function with a decreased glomerular filtration rate [22,23,24,25,26].

Nadir creatinine in the first year of life was an excellent predictor of CAKUT progression to ESRD. A cut-off of 0.98 mg/dL showed high sensitivity and specificity in identifying patients at risk of progression to ESRD. Katsoufis et al. utilized a threshold of 0.6 mg/dL for nadir creatinine in their study, which predicted the progression of CAKUT to ESRD with a sensitivity of 64% and a specificity of 93%. The AUC was 0.89. When using a threshold of 0.7 mg/dL, the sensitivity was 100%, and the specificity was 88% in predicting progression to CKD stages 3–5 [27]. Meneghesso et al. conducted a meta-analysis involving 13 articles and 1731 patients to evaluate the predictive significance of nadir creatinine levels in individuals with PUV. Across the studies, threshold values ranged from 0.7 to 2.7 mg/dL, with 1 mg/dL being commonly used as a benchmark. The meta-analysis concluded that a nadir creatinine level of 1 mg/dL was the most reliable prognostic indicator for renal function in individuals with PUV [28]. In another study on 96 patients with PUV, Coleman et al. showed that having a nadir creatinine level above 0.85 mg/dL was highly predictive of future chronic renal insufficiency. On the other hand, they concluded that a nadir creatinine below 0.4 mg/dL suggested a low risk of developing CKD [29]. Similar results were published by Luchita et al. They performed a study on 77 patients with PUV, finding that nadir creatinine was a reliable marker in predicting progression to CKD when a threshold of 0.85 mg/dL was used [30].

Another study that aimed to use serum creatinine value as an early prognostic factor in patients with CAKUT was conducted by Nishi et al. They performed a study on 92 patients with CAKUT, in which they evaluated the prognostic value of the maximum serum creatinine level in the first three days of life. Their results showed that a maximum value above 2.5 mg/dL was associated with a significantly lower kidney survival rate during infancy. Additionally, their study revealed that the maximum creatinine level in the first three days of life and oligohydramnios were associated with an increased risk of progression to infant renal failure requiring renal replacement therapy [31].

One important limitation of this study is the retrospective nature of the analysis. As a result, the patients were not followed up periodically according to a well-established protocol. As a result, some patients were only admitted to our service when they had already progressed to decompensated ESRD. Thus, we were unable to accurately determine the exact timing of disease progression for all patients, which limited our ability to evaluate the progression rate effectively. Another limitation of the study, due to its retrospective design, is the lack of a clear protocol for serial determination of serum creatinine values. This can lead to missing the lowest serum creatinine value in the first year of life. For an accurate assessment of serum creatinine dynamics, patients should be sampled daily, which is very challenging in children. This is likely one of the reasons why cut-off values determined by different studies vary. However, the differences are not very large, as specified above. These limitations highlight the need for future prospective studies to follow patients more systematically, allowing for a more precise assessment of the disease progression rate. Moreover, to assess the prognostic value of nadir creatinine with greater accuracy, prospective studies with larger patient cohorts and longer follow-up periods are needed.

## 5. Conclusions

In conclusion, our findings support the utility of a nadir creatinine cut-off of 0.98 mg/dL as a reliable predictor of progression to ESRD, consistent with previously published data. A nadir creatinine level exceeding the specified threshold in patients with CAKUT is indicative of an elevated risk of disease progression. This finding underscores the importance of establishing close coordination within a multidisciplinary team to implement strategies to decelerate the progression to ESRD. Such an approach enables comprehensive coordination of medical and surgical interventions to prevent infections, preserve and enhance residual renal function, and facilitate preemptive transplantation when feasible.

## Figures and Tables

**Table 1:** The mean gestational age at detection and the mean nadir creatinine for each type of malformation.

	Unilateral VUR	Bilateral VUR	PUV	UPJ	OU	MCDK
Gestational age (weeks)	30.06 ± 6.42	30.53 ± 5.19	27.03 ± 5.15	30.68 ± 7.16	30.62 ± 6.76	24.55 ± 7.17
Nadir creatinine (mg/dL)	0.43 ± 0.08	0.52 ± 0.26	0.62 ± 0.41	0.39 ± 0.04	0.48 ± 0.21	0.40 ± 0.04

## Data Availability

The data presented in this study are available on request from the corresponding author. The data are not publicly available due to privacy and ethical reasons.
